# Computed Tomography Imaging Findings of Pulmonary Chondroma

**DOI:** 10.1155/2018/4387689

**Published:** 2018-12-30

**Authors:** Hexiang Wang, Pei Nie, Cheng Dong, Feng Hou, Peng Zhang, Jizheng Lin, Jihua Liu

**Affiliations:** ^1^Department of Radiology, The Affiliated Hospital of Qingdao University, Qingdao 266003, Shandong, China; ^2^Department of Pathology, The Affiliated Hospital of Qingdao University, Qingdao 266003, Shandong, China; ^3^Department of Radiology, Capital Medical University, Beijing Friendship Hospital, Beijing 100000, China

## Abstract

**Purpose:**

To characterize the computed tomography (CT) imaging findings in patients with pulmonary chondroma.

**Methods:**

We examined CT imaging findings of eight patients with histopathologically verified pulmonary chondroma. We assessed the location, size, shape, margins, amount of calcification, calcification pattern, and attenuation on precontrast and enhancement CT.

**Results:**

All patients exhibited solitary, mildly lobulated pulmonary masses, which were located in the right lung in four cases and the left lung in four cases. The mean lesion size was 3.7 cm (range 0.9–10.7 cm). All eight tumours had a well-defined margin. On plain CT images, seven of the cases (87.5%) showed a mass with varying degrees of calcification, which included strip-like punctate (n=5) and ring (n=2) patterns. One patient with a large lesion (10.7 cm) showed chest wall adhesion. On contrast-enhanced CT images, all lesions demonstrated slight inhomogeneous enhancement ≤14 HU.

**Conclusion:**

CT is the reference standard diagnostic technique for locating pulmonary chondroma. In most cases, CT findings show some characteristics that are important in the diagnosis, surgical planning, and follow-up of the tumour.

## 1. Background 

Chondromas are benign tumours that originate from chondrocytes. Chondromas are typically found in the long bones and rarely arise in the lung parenchyma, with pulmonary chondroma only accounting for 0.04% of lung tumours [1]. Although the pulmonary chondroma is a benign condition, the association with Carney triad prompts further investigation [2]. The correct diagnosis for patients with Carney's triad is essential for determining the clinical management [3]. Therefore, accurate and comprehensive evaluation is very important to pulmonary chondroma. Because of the low associated morbidity, there are only a few reports of pulmonary chondroma [3–12], and the imaging features were not well examined. Thus, the aim of the present study was to determine the computed tomography (CT) imaging findings of pulmonary chondroma.

## 2. Methods

### 2.1. Patients

This study was approved by the institutional Medical Ethics Committee. Between May 2006 and January 2017, five female and three male patients (age range: 45–73 years, mean age: 55.6 years) with pathologically proven pulmonary chondroma were retrospectively analysed. All eight patients had undergone surgery for the disease. Four of the eight patients exhibited a cough, two of whom showed haemoptysis and shortness of breath, one patient exhibited chest pain as the predominant symptom, and three patients had no clinical symptoms.

CT was performed using a 16-slice to 128-slice multidetector (GE light speed 16 slice; GE bright speed 16 slice; Somatom Sensation 64 slice; Discovery CT 750 HD 128 slice). Seven patients underwent plain axial CT scanning using a soft tissue algorithm. CT images were acquired in the arterial (25–30 s after injection) and venous (50–60 s after injection) phases in six patients.

### 2.2. Imaging Analysis

Two radiologists (7 years of experience in chest radiology) retrospectively evaluated the images and independently reviewed the tumour location (the lobe), size, shape (circular or lobulated), margins (well-defined or ill-defined), amount of calcification, calcification pattern, and attenuation (homogeneous or inhomogeneous and CT value) on precontrast and enhancement CT. To determine the amount of calcification within each mass, a subjective scoring system of 1–3 was used (<20%, 20-50%, and >50% calcification in the mass). A score of 1 was assigned to minimal calcification, and scores of 2 and 3 were assigned to moderate and severe calcification, respectively. The calcification pattern included strip-like punctate, sheet, ring, round, and irregular. The attenuation values of the tumours were measured in each mass/nodule in precontrast CT and all two enhancement phases of imaging in the exact same location in each mass/nodule to acquire the attenuation. Each lesion was measured three times and the average attenuation value was used for recording. The enhancement value=the attenuation value in enhancement CT-the attenuation value of precontrast CT. The size of each lesion was measured at its greatest single dimension. Other associated lesion satellites and enlarged lymph nodes at the mediastinum or hilus of the lung were also recorded.

## 3. Results

**Figure 1:**
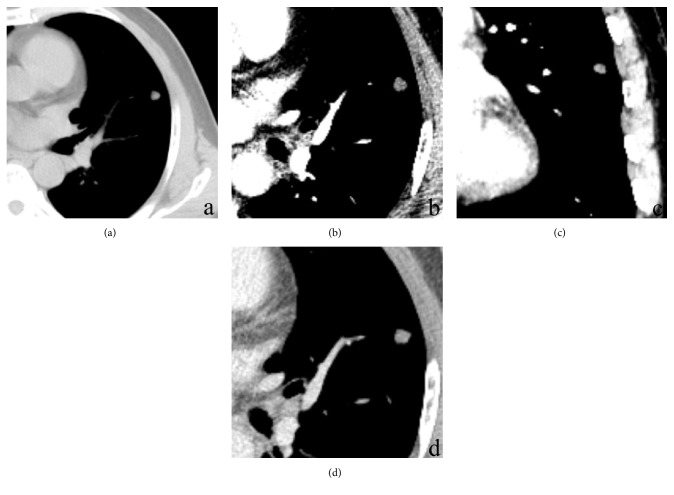
A 62-year-old woman with pulmonary chondroma. Mediastinal window images are shown. Computed tomography (CT) shows a slightly lobulated, well-defined nodule in the left upper lobe of the lung. (a) Precontrast axial CT scan shows an inhomogeneous soft tissue density nodule (19 HU). (b) Postcontrast axial arterial phase scan. (c) Postcontrast coronal arterial phase scan. (d) Postcontrast axial venous phase scan. The lesion shows a slight enhancement of 10 HU (arterial) and 14 HU (venous).

**Figure 2:**
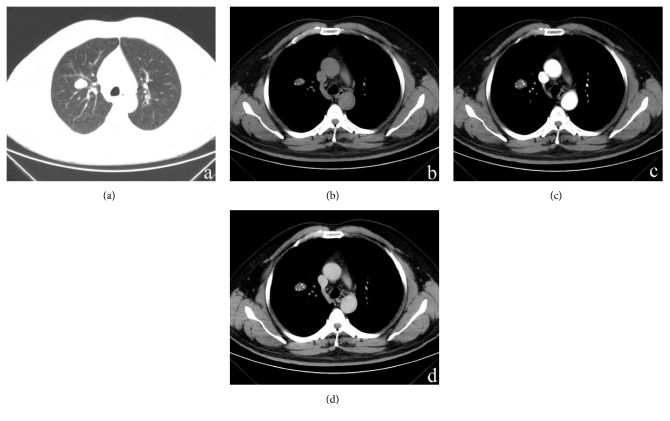
A 47-year-old woman with pulmonary chondroma. (a) Axial view and (b–d) lung window mediastinal window images. CT shows a mildly lobulated, well-defined nodule with strip-like, punctate calcification in the right upper lobe of the lung. (b) Precontrast scan shows an inhomogeneous soft tissue density nodule (22 HU). (c) Postcontrast arterial phase scan. (d) Postcontrast venous phase scan. The lesion shows a slight enhancement of 3 HU (arterial) and 8 HU (venous).

**Figure 3:**
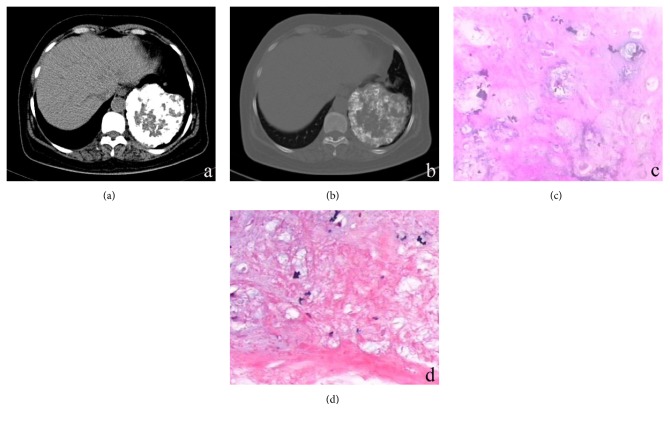
A 53-year-old woman with pulmonary chondroma. Axial view (a) and (b) mediastinal, bone-window images. CT shows a slightly lobulated, well-defined mass with a characteristic ring calcification in the left lower lobe of the lung. The patient with a large lesion (10.7 cm) showed chest wall adhesion suggestive of malignant infiltration. (c, d) The lesion was comprised of mature myxoid cartilage and calcification cartilage (Haematoxylin and Eosin; 400X magnification).

The clinical and CT features displayed by the eight patients with pulmonary chondroma are shown in Table 1. All patients exhibited solitary pulmonary masses (Figures 1–3). All lesions exhibited mild lobulation (Figures 1–3). The mean lesion size was 3.7 cm (range, 0.9–10.7 cm). All eight tumours had a well-defined margin without glitches.

On plain CT images, seven of the cases (87.5%) showed a mass with varying degrees of calcification, which included five masses with strip-like punctate patterns defined as scoring 1 (Figure 2) and two lesions with ring (Figure 3) patterns defined as scoring 3. One patient with a large lesion (10.7 cm) showed chest wall invasion. Only one patient with a smaller lesion (0.9 cm) displayed no calcification (Figure 1). The mean arterial enhancement value was 6.8 HU (range, 3–10 HU). The mean venous enhancement value was 9.8 HU (range, 8–14 HU). On contrast-enhanced CT images, all lesions demonstrated a slight inhomogeneous enhancement ≤14 HU (Figures 1 and 2). No satellite lesions or significant enlargement of the lymph nodes or pulmonary cavity were observed.

Pathological examination showed that the tumours were composed of mature myxoid cartilage and calcification cartilage, with a fibrous pseudocapsule surrounding the tumour (Figures 3(c) and 3(d)).

## 4. Discussion

The origin of pulmonary chondroma remains unclear [8]. During embryonic development, pulmonary chondroma often originates from the ectopic cartilage of the lung tissue, while chondrocytes within the bloodstream can also flow into the lungs. Under certain conditions, reticulocytes and connective tissues developing into original directions become the embryo of mesenchymal tissue and then develop into chondrocytes [8].

Pulmonary chondroma was reported to commonly occur in adult women at 40–50 years of age [8]. The present study had a cohort of five women and three men with pulmonary chondroma (average age, 55.6 years; range, 45–73 years) [8, 10, 13]. Pulmonary chondroma can occur in the pulmonary lobes, particularly in the right lung [3]. By contrast, we found an even distribution of chondroma in the left (four cases) and right (four cases) lobes. In general, in absence of symptoms in most patients [14], the symptoms of pulmonary chondroma depend on the size and location of the tumour. Respiratory symptoms, including cough with haemoptysis or sputum and shortness of breath, are often observed [7]; this occurred in four cases (50%) in the present study. These symptoms are mainly due to the compression of the tumour on surrounding tissues. Chest pain was also reported in patients with pulmonary chondroma that involved the intercostal nerves [15]. In the present study, one patient with a large tumour and chest wall invasion exhibited an exact symptom chest pain.

The CT imaging appearance of pulmonary chondroma depends on the location and extent of involvement. According to our findings and previous reports, the typical CT features of pulmonary chondroma include (1) round or oval masses with clear boundaries, (2) lesion size ranging from 1.0 to 4.0 cm, with slight lobulation [3], (3) inhomogeneous soft tissue density mass with calcification [3–6, 10], and (4) no glitches, satellite lesions, or enlarged lymph nodes at the hilus of lung or mediastinum [3, 6].

There are no reported CT findings specific for identification of pulmonary chondroma. In the present study, five of eight pulmonary chondromas were nodules (<3 cm diameter). A number of studies have suggested that CT findings of a smooth border, benign calcification (central, popcorn, laminated, or diffuse), and enhancement ≤15 HU may indicate benign nodules [16–20], which is consistent with our findings, except for the calcification patterns. There are six reported calcification patterns in pulmonary nodules: (1) central dense nidus, (2) diffuse solid, (3) popcorn, (4) laminated, (5) punctate, and (6) dendriform; [21] patterns 1–4 represent benign calcifications. In the present study, four patients exhibited punctate calcific (defined as scoring 1) lung nodules, which is regarded as a sign of malignancy [22, 23]. Thus, a benign nodule imaging feature with a malignant calcification pattern is highly suggestive of a diagnosis of pulmonary chondroma. We also observed one case with imaging features (without calcification) consistent with a benign nodule, which is not diagnostic for pulmonary chondroma.

In the present study, there were three cases with pulmonary chondroma >3 cm with punctate (n=1, defined as scoring 1) or characteristic ring (n=2, defined as scoring 3) calcifications. These smooth border, mildly lobulated masses are difficult to diagnose. However, characteristic ring calcifications may be suggestive of pulmonary chondroma.

Chondroma can also display no or slight early enhancement and delayed enhancement after delivery of contrast agents, because of the relatively slow blood flow within the tumour [24, 25]. In our study, all five patients showed contrast-enhanced CT enhancement ≤14 HU. Thus, this imaging feature can provide valuable information on the blood supply to the tumour and help predict whether it is benign.

Pulmonary chondroma is also one of the clinical manifestations of Carney's triad, which involves the presence of at least two components of pulmonary chondroma, gastrointestinal stromal tumours, and extra-adrenal paraganglioma [26]. The correct diagnosis for patients with Carney's triad is essential for determining the clinical management [3]. Recent studies also suggest that chondromas in the Carney's triad mainly affect young women and are small, multiple, peripheral, and commonly calcified masses [2, 9–11, 27]. As gastrointestinal stromal tumours and extra-adrenal paraganglioma are potentially fatal, such patients should receive further examination to check for the presence of other components of this rare syndrome [28, 29]. Although pulmonary chondromas are benign tumours of cartilage, there is also potential for malignant transformation. Indeed, a case of giant pulmonary chondrosarcoma with tumour recurrence and metastasis was reported [30]. Further, patients with Carney's triad can exhibit malignant alterations of masses. Thus, patients with Carney's triad should receive regular medical follow-up.

Because of its low morbidity, and as patients are often asymptomatic, pulmonary chondroma can be easily misdiagnosed as tuberculosis tumours, hamartoma (particularly cartilage hamartoma), and peripheral lung cancer. Indeed, patients with a previous history of tuberculosis were commonly diagnosed with suspected pulmonary tuberculoma [31]. Tuberculoma is often located in the superior lobe apicoposterior segment or the lower lobe dorsal segment of the lung. Kim et al. [18] also reported that presence of cavitation and satellite lesions on chest CT is useful for tuberculoma diagnosis. Hamartoma is the most common benign lung tumour, accounting for 5%–10% of solitary pulmonary nodules [32]. The majority of patients with pulmonary hamartomas are men, while pulmonary chondroma predominantly occurs in adult women. The identification of fat and calcification is a specific feature for hamartoma [21]; on CT, fat was detected in 34%–50% of hamartoma cases, while calcification was only observed in 15%–30% of cases. By contrast, seven of eight (87.5%) of our cases with pulmonary chondroma exhibited calcification, and all cases had an absence of fat. Hamartoma usually shows a popcorn calcification on CT, which is used for differential diagnosis. However, in the present study the pulmonary chondroma calcifications were often smaller, with punctate or ring characteristics. For older patients who perennially smoke, a cough with sputum and blood can also be easily misdiagnosed as peripheral lung cancer. Peripheral lung cancer usually displays a burr-like tumour structure, pleural indentation, and inhomogeneous enhancement, often accompanied by enlarged lymph nodes at the hilus of the lung or mediastinum [33]. However, a study of 500 CT examinations suggested that lung carcinoma calcifications are typically large and located centrally in tumours [23].

In conclusion, the preoperative diagnosis of pulmonary chondroma is difficult, and a variety of imaging techniques are required for comprehensive diagnosis. Using CT, we found that lesions showing a solitary, mildly lobulated mass with a well-defined margin, calcification (punctate, strip-like, or ring calcifications), and slight enhancement (<15 HU) should be considered potential pulmonary chondroma. Nevertheless, differential diagnosis of hamartoma (particularly cartilage hamartoma), tuberculosis tumour, and peripheral lung cancer is also important. Further clinical, imaging, and pathological studies are required to improve the diagnosis of this disease. After diagnosis of pulmonary chondroma, further examination is also required to exclude Carney's triad.

## Figures and Tables

**Table 1:** Clinical and CT imaging findings in eight patients with an pulmonary chondroma.

Patient/age/gender	Location	Maximum diameter (cm)	Shape	Margin	Attenuation (plain CT)	Calcification Pattern and grade	Enhancement Attenuation (arterial)	Enhancement attenuation (venous)	Enhancement pattern
1/57/F	right middle lob	5.4	mild lobulated	well-defined	39HU	Ring/ 3			
2/49/F	right lower lobe	2.9	mild lobulated	well-defined	25HU	strip-like, punctate/ 1	31HU EV=6HU	37HU EV =12HU	inhomogeneous enhancement
3/45/M	left lower lobe	4.2	mild lobulated	well-defined	27HU	strip-like, punctate/ 1	36HU EV =9HU	41HU EV =14HU	inhomogeneous enhancement
4/59/M	right upper lobe	1.3	mild lobulated	well-defined	21HU	strip-like, punctate/ 1	27HU EV =6HU	32HU EV =11HU	inhomogeneous enhancement
5/73/M	left lower lobe	1.1	mild lobulated	well-defined		strip-like, punctate/ 1	29HU	35HU	inhomogeneous enhancement
6/62/F	left upper lobe	0.9	mild lobulated	well-defined	19HU		29HU EV =10HU	33HU EV =14HU	inhomogeneous enhancement
7/47/F	right upper lobe	2.8	mild lobulated	well-defined	22 HU	strip-like, punctate/ 1	25HU EV =3 HU	30HU EV =8 HU	inhomogeneous enhancement
8/53/F	left lower lobe	10.7	mild lobulated	well-defined	43HU	Ring/ 3			

**Note:** EV: enhancement value=the attenuation value in enhancement CT-the attenuation value of precontrast CT.

## Data Availability

The data used to support the findings of this study are available from the corresponding author upon request.

## References

[1] Bateson E. M. (1970). Histogenesis of intrapulmonary and endobronchial hamartomas and chondromas (cartilage‐containing tumours): A hypothesis. *The Journal of Pathology*.

[2] Shi G., Cui Y., He Y., Gong M. (2016). An unusual case of incomplete Carney triad: An 18-year-old girl suffering from multiple benign tumors. *Journal of Thoracic Disease*.

[3] Tian D., Wen H., Zhou Y., Fu M. (2016). Pulmonary chondroma: A clinicopathological study of 29 cases and a review of the literature. *Molecular and Clinical Oncology*.

[4] Yang G., Wang X., Wang Z., Jiang Y., Fu J. (2011). Tc-99m MDP Uptake in a Giant Pulmonary Chondroma. *Clinical Nuclear Medicine*.

[5] Mydin H. H., Kerr K. M., Dempsey O. (2014). Calcified pulmonary chondromas in Carney's triad. *Thorax*.

[6] Strano S., Ouafi L., Baud M., Alifano M. (2010). Primary Chordoma of the Lung. *The Annals of Thoracic Surgery*.

[7] Ammar A., El Hammami S., Sellami Kamoun N. (2005). Sellami Kamoun, [Chondromas - a rare lung tumour]. *Revue des Maladies Respiratoires*.

[8] Hoekstra M. O., Bertus P. M., Nikkels P. G. J., Kimpen J. L. L. (1994). Multiple pulmonary chondromata: A rare cause of neonatal respiratory distress. *CHEST*.

[9] Spatz A., Bressac-De Paillerets B., Raymond E. (2004). Case 3. Gastrointestinal stromal tumor and Carney's triad. *Journal of Clinical Oncology*.

[10] Rodriguez F. J., Aubry M.-C., Tazelaar H. D., Slezak J., Aidan Carney J. (2007). Pulmonary chondroma: a tumor associated with carney triad and different from pulmonary hamartoma. *The American Journal of Surgical Pathology*.

[11] Qiao G.-B., Zeng W.-S., Peng L.-J. (2009). Multiple pulmonary chondromas in a young female patient: a component of carney triad. *Journal of Thoracic Oncology*.

[12] Papadakis G. Z., Patronas N. J., Chen C. C., Carney J. A., Stratakis C. A. (2015). Combined PET/CT by 18F-FDOPA, 18F-FDA, 18F-FDG, and MRI correlation on a patient with Carney triad. *Clinical Nuclear Medicine*.

[13] Carney J. A. (1999). Gastric stromal sarcoma, pulmonary chondroma, and extra-adrenal paraganglioma (Carney triad): natural history, adrenocortical component, and possible familial occurrence. *Mayo Clinic Proceedings*.

[14] Chen C., Chuang C., Liu M., Hsu W., Lin H., Hsieh J. (2010). Clinical, radiologic and pathologic characteristics of the carney triad: a case report and literature review. *Kaohsiung Journal of Medical Sciences*.

[15] Allan J. S. (2003). Rare solitary benign tumors of the lung. *Seminars in Thoracic and Cardiovascular Surgery*.

[16] Winer-Muram H. T. (2006). The solitary pulmonary nodule. *Radiology*.

[17] Henschke C. I., Yankelevitz D. F., Mirtcheva R., McGuinness G., McCauley D., Miettinen O. S. (2002). CT screening for lung cancer: Frequency and significance of part-solid and nonsolid nodules. *American Journal of Roentgenology*.

[18] Kim H. J., Kang S. J., Suh G. Y. (2001). Predictors for benign solitary pulmonary nodule in tuberculosis - endemic area. *The Korean Journal of Internal Medicine*.

[19] Kuriyama K., Tateishi R., Doi O. (1991). Prevalence of air bronchograms in small peripheral carcinomas of the lung on thin-section CT: Comparison with benign tumors. *American Journal of Roentgenology*.

[20] Zwirewich C. V., Vedal S., Miller R. R., Müller N. L. (1991). Solitary pulmonary nodule: high-resolution CT and radiologic-pathologic correlation. *Radiology*.

[21] Khan A. N., Al-Jahdali H. H., Allen C. M., Irion K. L., Al Ghanem S., Koteyar S. S. (2010). The calcified lung nodule: What does it mean. *Annals of Thoracic Medicine*.

[22] Mahoney M. C., Shipley R. T., Corcoran H. L., Dickson B. A. (1990). CT demonstration of calcification in carcinoma of the lung. *American Journal of Roentgenology*.

[23] Grewal R. G., Austin J. H. M. (1994). Ct demonstration of calcification in carcinoma of the lung. *Journal of Computer Assisted Tomography*.

[24] Thompson J. E., Castillo M., Thomas D., Smith M. M., Mukherji S. K. (1997). Radiologic-pathologic correlation polymicrogyria. *American Journal of Neuroradiology*.

[25] Duan F., Qiu S., Jiang J. (2012). Characteristic CT and MRI findings of intracranial chondroma. *Acta Radiologica*.

[26] Carney J. A., Sheps S. G., go V. L. W., Gordon H. (1977). The Triad of Gastric Leiomyosarcoma, Functioning Extra-Adrenal Paraganglioma and Pulmonary Chondroma. *The New England Journal of Medicine*.

[27] Vega J., Navarro Subiabre J., Lovera Riquelme C., Opazo H., Santamarina M. (2017). Carney triad. Report of one case. *Revista Médica de Chile*.

[28] MARGULIES K. B., SHEPS S. G. (1988). Carney's Triad: Guidelines for Management. *Mayo Clinic Proceedings*.

[29] Ishii H., Akiba T., Marushima H., Kanetsuna Y., Morikawa T. (2012). A case of bilateral multiple pulmonary chondroma: Necessity of follow-up for Carney's triad. *General Thoracic and Cardiovascular Surgery*.

[30] Mei B., Lai Y.-L., He G.-J., Shou Y.-N., Liu J. (2013). Giant primary mesenchymal chondrosarcoma of the lung: Case report and review of literature. *Annals of Thoracic and Cardiovascular Surgery*.

[31] Fain O. (2005). [Pulmonary tuberculoma]. *La Revue du Praticien*.

[32] Dragoumis D. M., Boudalaki E. S., Assimaki A. S., Tsiftsoglou A. P. (2012). Pulmonary hamartoma masquerading lung metastasis in a woman with inflammatory breast cancer. *The Breast Journal*.

[33] Yokota H., Shigeta A., Edo H., Niijima M. (2007). Peripheral lung cancer effectively diagnosed with virtual bronchoscopy. *Nihon Naika Gakkai zasshi. The Journal of the Japanese Society of Internal Medicine*.

